# 

**Published:** 1843-07

**Authors:** 


### CONTENTS OF No. XXXI.

OF THE

### British and Foreign Medical Review.

JULY, 1843.

---

### PART FIRST.—Analytical and Critical Reviews.

PAGE

ART. I.—Traité du Ramollissement du Cerveau. Par MAX. DURAND-FARDEL, M.D. &c. &c. . . . . . . 1

Treatise on Softening of the Brain. By MAX. DURAND-FARDEL, M.D. &c. &c.

ART. II.—The Climate of the United States and its Endemic Influences, based chiefly on the Records of the Medical Department and Adjutant-General's Office, United States Army. By SAMUEL FORRY, M.D. . . 34

ART. III.—Geschichtliche Darstellung der grösseren chirurgischen Operationen mit besonderer Rucksicht auf Edlen von Wattmann's Operations-Methoden. Von Dr. FERDINAND HEBRA . . . . . . 46

An Historical Account of the more important Operations in Surgery with especial reference to the mode employed by Dr. Von Wattmann. By FERDINAND HEBRA, M.D.

ART. IV.—Handbuch der gerichtlichen Medicin, &c. für Aerzte und Criminalisten. Von Dr. G. H. NICOLAI . . . . . 68

A Manual of Medical Jurisprudence, for Physicians and Jurists. By Dr. G. H. NICOLAI.

ART. V.—1. On the different forms of Insanity in relation to Jurisprudence. By JAMES COWLES PRICHARD, M.D. . . . . . 81

2. The Plea of Insanity in Criminal Cases. By FORBES WINSLOW, M.R.C.S. . ib.

3. Criminal Jurisprudence considered in relation to Cerebral Organization. By M. B. SAMPSON . . . . . . ib.

§

A 293

PAGE

4. Commentaries on some Doctrines of a dangerous tendency in Medicine. (Comm. III.—On some Important Questions relating to Insanity, both in a Medical and Legal point of view.) By SIR ALEX. CRICHTON, M.D. F.R.S. . 81

5. Report of the Trial of Daniel M'Naughten for the wilful Murder of Edward Drummond, Esq. By R. M. BOUSFIELD and RICHARD MERRETT . ib.

6. M'Naughten. A Letter to the Lord Chancellor upon Insanity. By J. Q. RUMBALL, Esq. M.R.C.S. . . . . . . ib.

7. On the Amendment of the Law of Lunacy; a Letter to Lord Brougham. By a Phrenologist . . . . . . ib.

ART. VI.—Mémoire sur le Glaucôme. Par le Docteur JULES SICHEL . . 110

Memoir on Glaucoma. By Dr. J. SICHEL.

ART. VII.—The Life of Sir Astley Cooper, Bart., interspersed with Sketches from his Note-books of distinguished contemporary characters. By BRANSBY B. COOPER, Esq. F.R.S. . . . . . . 118

ART. VIII.—Chemie und Medicine in ihrem engeren Zusammenwirken, oder Beduetung der neuren Fortschritte der organischen Chemie für erfahrungsmässige und speculative ärztliche Forschung, als vollständige Lehrschrift für die studien der organischen Chemie uberhaupt, insbesondere aber für die im Gebiete der Medicin und Pharmacie, so wie für die Fortschritte der Heilmittellehre. Von Dr. F. L. HUENEFELD . . . . 145

Chemistry and Medicine in close cooperation; or a view of the latest progress of Organic Chemistry in relation to experimental and speculative medical research, intended as a complete text-book for the study of organic chemistry generally, and particularly in the domain of Medicine and Pharmacy, as also Materia Medica. By Dr. F. L. HUENEFELD.

ART. IX.—Friederich Tiedemann, Professor in Heidelberg, von den Duverneyschen Bartholinschen, oder Cowperschen Drüsen des Weibs und der schiefen Gestaltung und Lage der Gebärmutter . . . . 155

Professor TIEDEMANN on the Glands of Duverney, Bartholinus, or Cowper in the Human Female; and on Obliquity in the form and position of the Uterus.

ART. X.—1. Die Lehre von der Reflex-Function, für Physiologen und Aerzte, dargestellt und beurtheilt von JOHANN WILHELM ARNOLD, M.D. . . 158

The Doctrine of the Reflex-Function, in its relations to Physiology and Practical Medicine, displayed and critically examined by Dr. J. W. ARNOLD.

2. On the Diseases and Derangements of the Nervous System, in their primary forms, and in their modifications by Age, Sex, Constitution, Hereditary Predisposition, Excesses, General Disorder, and Organic Disease. By MARSHALL HALL, M.D. F.R.S. L. and E. &c. &c. . . . ib.

PAGE

ART. XI.—Ueber die Veränderungen des Scheidentheiles und der unteren Abschnittes der Gebärmutter in der zweiten Hälfte der Schwangerschaft. Von Dr. Fr. H. G. BIRNBAUM . . . . . . 184

An Essay on the Changes which the Cervix and Lower Segment of the Uterus undergo in the second half of Pregnancy. By Dr. F. H. G. BIRNBAUM.

ART. XII.—Pharmacologia; being an extended Inquiry into the Operations of Medicinal Bodies, upon which are founded the Theory and Art of Prescribing. By J. A. PARIS, M.D. Cantab. F.R.S. &c. . . . . 188

ART. XIII.—Report of the Committee appointed by the Right Hon. the Governor of Bengal for the establishment of a Fever Hospital, and for inquiring into Local Management and Taxation in Calcutta . . . 206

ART. XIV.—Ueber die Verjungung des Menschlichen Lebens und die Mittel und Wege zu ihrer Cultur. Nach physiologischen Untersuchungen in praktischer Anwendung dargestelt von Dr. CARL HEINRICH SCHULTZ, &c. . . 208

On the Rejuvenescence of Man, or Renewal of Human Life, and on the Means and Modes of cultivating it; a practical Treatise, founded on Physiological Researches. By Dr. C. H. SCHULTZ.

ART. XV.—Solution du Problème de la Population et de la Subsistance, &c. Par CHARLES LOUDON . . . . . . 229

A Solution of the Problem of Population and Subsistence; in a Series of Letters to a Physician. By CHARLES LOUDON, M.D. &c.

ART. XVI.—A System of Clinical Medicine. By R. J. Graves, M.D. M.R.I.A. . 232

ART. XVII.—Medical History of the Expedition to the Niger during the years 1841-2, comprising an account of the Fever which led to its abrupt termination. By J. O. M'WILLIAM, Surgeon of H. M. S. Albert, and senior medical officer of the Expedition . . . . . 259

---

### PART SECOND.—Bibliographical Notices.

ART. I.—The Life of a Travelling Physician, from his first introduction to Practice, including Twenty years' Wanderings through the greater part of Europe . 265

ART. II.—Du Bonheur en Chirurgie, Recueil de Faits Cliniques. Par J. MOULINIÉ, Ex-Chirurgien en chef de l'Hôpital de Bourdeaux, Professeur de Clinique Chirurgicale, &c. . . . . . . 266

Success in Surgery; a Collection of Clinical facts. By J. MOULINÉ, late Principal Surgeon to the Hospital of Bourdeaux, Professor of Clinical Surgery, &c.

PAGE

ART. III.—Beiträge zur Pathologie und Therapie mit besonderer Berucksichtigung der Chirurgie von DR. CARL EMMERT . . . . 267

Contributions to Pathology and Therapeutics, with especial reference to Surgery. By CHARLES EMMERT, M.D.

ART. IV.—Jahresbericht über die Fortschritte der gesammten Medicin in allen Ländern. Herausgegeben von Dr. C. CANSTATT . . . 268

Annual Report on the Progress of Medicine in general in all Countries. Edited by Dr. C. CANSTATT.

ART. V.—Beobachtungen über den Nützen und Gebrauch des Keilschen Magnet-Electrischen Rotations apparats in Krankheiten, &c. Von J. E. WETZLER . . . . . . . 269

Observations on the Use of Professor Keil's Magneto-Electric Apparatus in various diseases, especially in Chronic Neuroses, Rheumatic, and Gouty Affections. By J. E. WETZLER.

ART. VI.—Clinical Remarks on certain Diseases of the Eye, and on Miscellaneous Subjects, Medical and Surgical, including Gout, Rheumatism, Fistula, Cancer, Hernia, Indigestion, &c. &c. By JOHN CHARLES HALL, M.D., of East Retford . . . . . . . 270

ARL. VII.—An Essay on the Nature and Treatment of Apoplexy. By M. GAY. Translated by EDWARD COPEMAN, Surgeon; with an Appendix . . 272

---

APPENDIX to Article V. p. 81, on the Medical Jurisprudence of Insanity . 273

---

### PART THIRD.—Original Reports and Memoirs.

Report on the New Test for Arsenic, and its value compared with the other methods of detecting that poison. By ALFRED S. TAYLOR . . 275

---

### PART FOURTH.—Medical Intelligence.

1. On the Neutral Azotized Materials of Organization. By MM. DUMAS and CAHOURS . . . . . . . 283

2. Researches on the quantity of Carbonic Acid exhaled from the Lungs of the Human Species. By MM. ANDRAL and GAVARRET . . . 285

3. Blood-corpuscles and Spermatozoa of the Camelidæ . - . 287

4. Blood-corpuscles of the Stanley Musk Deer . . . . ib.

5. BOOKS RECEIVED FOR REVIEW . . . . . . . ib.

